# Age-related bone turnover markers and osteoporotic risk in native Chinese women

**DOI:** 10.1186/1472-6823-14-8

**Published:** 2014-01-22

**Authors:** Xi-Yu Wu, Hong-Li Li, Hui Xie, Xiang-Hang Luo, Yi-Qun Peng, Ling-Qing Yuan, Zhi-Feng Sheng, Ru-Chun Dai, Xian-Ping Wu, Er-Yuan Liao

**Affiliations:** 1 Institute of Metabolism and Endocrinology, The Second Xiangya Hospital, Central South University, No.139 Middle Renmin Road, Changsha, Hunan 410011, People’s Republic of China; 2 Department of Endocrinology, The First Hospital, Lanzhou University, No.1 West Donggang Road, Lanzhou, Gansu 730000, People’s Republic of China

**Keywords:** Bone turnover markers, BMD T-scores, Osteoporosis, Osteoporotic risk, Native Chinese women

## Abstract

**Background:**

The rate of bone turnover is closely related to osteoporosis risk. We investigated the correlation between bone turnover markers and BMD at various skeletal sites in healthy native Chinese women, and to study the effect of changes in the levels of bone turnover markers on the risk of osteoporosis.

**Methods:**

A cross-section study of 891 healthy Chinese women aged 20–80 years was conducted. The levels of serum osteocalcin (OC), bone-specific alkaline phosphatase (BAP), serum cross-linked N-terminal telopeptides of type I collagen (sNTX), cross-linked C-terminal telopeptides of type I collagen (sCTX), urinary NTX (uNTX), urinary CTX (uCTX) and total urinary deoxypyridinoline (uDPD) were determined. BMD at the posteroanterior spine and the hip was measured using DXA.

**Results:**

Pearson’s correlation coefficient found significant negative correlation between bone turnover marker and BMD T-score at different skeletal sites (*r* = −0.08 to −0.52, all *P* = 0.038–0.000). After adjustments for age and body mass index, the partial correlation coefficients between the OC, BAP, sNTX, sCTX and uCTX, and the T-scores at various skeletal sites were still significant. After adjustment of height and weight, the correlation coefficients between most BTMs and PA lumbar spine BMD were also significant. Multiple linear regression analysis showed that bone turnover markers were negative determinants of T-scores. BAP and OC accounted for 33.1% and 7.8% of the variations in the T-scores of the PA spine, respectively. Serum OC, BAP, uDPD, and sNTX accounted for 0.4–21.9% of the variations in the femoral neck and total hip T-scores. The bone turnover marker levels were grouped as per quartile intervals, and the T-scores, osteoporosis prevalence and risk were found to markedly and increase with increase in bone turnover marker levels.

**Conclusions:**

This study clarified the relationship between bone turnover markers and osteoporosis risk in native Chinese women. Bone turnover marker levels were found to be important determinants of BMD T-scores. Furthermore, osteoporotic risk significantly increased with increase in the levels of bone turnover markers.

## Background

Osteoporosis is a common disease, especially in women. In the USA, 55% of adults aged over 50 years have osteoporosis, with women accounting for approximately 80% of osteoporotic patients [1]. In Japan, approximately 49% of women aged over 50 years have osteoporosis [2], whereas in China, an estimated 88 million patients have primary osteoporosis [3]. Thus, primary osteoporosis is a considerable public health problem in the elderly. Increase in the rate of bone turnover is closely related to an increase in the risk of primary osteoporosis [4], and bone turnover markers can identify the rate of ongoing bone turnover. Many studies have shown a correlation between increased rates of bone loss and increased levels of bone turnover markers [5-16]. Moreover, changes in bone turnover markers can predict postmenopausal bone loss [5,12,17,18] and the risk of osteoporotic fractures [18-22], especially trabecular bone fractures [21]. Thus, bone turnover markers are clinically significant, as they can predict bone loss and osteoporotic risk, help monitor osteoporosis treatment, and clarify the therapeutic effect of resistance to absorption [22-24]. However, regional and ethnic differences have been found in the levels of bone turnover markers and rates of bone loss in women [25,26]. Furthermore, the relationship of bone turnover markers with bone mineral density (BMD) T-scores and the risk and prevalence of osteoporosis has not been clarified in native Chinese women. In order to determine the nature of this relationship in native Chinese women of different age groups, we adopted a cross-sectional study design. In total 891 healthy women living in the central and southern areas of China underwent tests for bone formation markers, including serum bone-specific alkaline phosphatase (BAP) and serum osteocalcin (OC), and bone resorption markers, including serum cross-linked N-terminal telopeptides of type I collagen (sNTX), serum cross-linked C-terminal telopeptides of type I collagen (sCTX), urinary NTX (uNTX), urinary CTX (uCTX) and total urinary deoxypyridinoline (uDPD). They also underwent dual energy X ray absorptiometry to determine BMD of the posteroanterior aspect of the lumbar vertebrae and proximal femur.

## Methods

### Study participants

We randomly selected 891 healthy Chinese women, aged 20–80 years, between May 2007 and October 2010. All the women were residents of Changsha and its surrounding regions and were recruited by public health organizations (i.e., health stations/clinics) that provide health care for the local residents. All subjects were screened using a detailed questionnaire, history, and physical examination. Subjects were excluded from the study if they had conditions that affect bone metabolism, such as diseases of the kidney, liver, parathyroid, and thyroid, diabetes mellitus, oligomenorrhea or menopause before the age of 40 years, hyperprolactinemia, oopherectomy, rheumatoid arthritis, ankylosing spondylitis, malabsorption syndromes, malignant tumors, hematological diseases, or previous pathological fractures. Subjects were also excluded if they were being treated with glucocorticoids, estrogens, thyroid hormone, fluorides, bisphosphonates, calcitonin, thiazide diuretics, barbiturates, anti-seizure medications, vitamin D, or calcium-containing drugs. Of the 891 study subjects, 490 were premenopausal, and 401 were postmenopausal (last menses >12 months before the beginning of the study). In the latter group, the mean age at menopause was 48.0 years (range, 40–57 years), and the median duration of menopause was 9.0 years (range, 1–38 years). The study was approved by the ethics committee of Xiang-Ya Medical College, Central South University (China), and all participants provided written consent to participate.

Blood samples were collected between 7 a.m. and 9 a.m. after an overnight fast and used to measure the serum levels of OC, BAP, sNTX and sCTX. Second morning urine samples were used to measure uNTX, uCTX, total uDPD and creatinine (Cr). Both serum and urine samples were stored at −70°C until assayed.

### Marker measurements

Enzyme-linked immunosorbent assay (ELISA) kits were used to measure the serum concentrations of BAP (Metra™ BAP EIA kit, Quidel Corporation, San Diego, CA, USA), OC (Diagnostic Systems Laboratories Inc., Webster, TX, USA), sNTX and uNTX (Osteomark, Ostex International Inc., Seattle, WA, USA), sCTX and uCTX (Nordic Bioscience Diagnostics A/S, Herlev, Denmark), and uDPD (Metra™ DPD EIA kit, Quidel Corporation, San Diego, CA, USA). The concentrations of urinary Cr were measured using an Auto Biochemistry Analyzer 7170A (Hitachi Co. Ltd., Tokyo, Japan). The other markers of bone turnover were measured using a μQuant microplate spectrophotometer (BIO-TEK Instruments Inc., Highland Park, Winooski, VT, USA). The levels of uNTX, uCTX and uDPD were corrected using the urinary Cr level. The sNTX level was expressed as nanomoles of bone collagen equivalents (nM BCEs) per liter, and the uNTX level was expressed as nM BCE per millimole creatinine (mM Cr) per liter. The intra- and inter-assay coefficients of variation (CVs) in order were as follows: 5.1% and 7.0% for BAP, 5.7% and 7.7% for OC, 4.6% and 6.9% for sNTX, 5.3% and 7.9% for sCTX, 6.1% and 7.6% for uNTX, 5.6% and 7.1% for uCTX, and 5.9% and 6.5% for uDPD [27].

BMD was measured with a DXA fan-beam bone densitometer (Hologic Delphi A; Hologic, Bedford, MA, USA) at various skeletal sites, including the PA lumbar spine (L1–L4), the femoral neck, and total hip. The in vivo precision deviations between two repeated BMD measurements at different skeletal sites in 33 subjects were determined by the root-mean-square CV method [28] and were 0.83% for the PA lumbar spine, 1.17% for the femoral neck, and 0.88% for the total hip. The control spine phantom scan performed each day had a long-term (>14 years) CV of <0.45%.

### Statistical analysis

All calculations were performed using SPSS V17.0 for Windows software (SPSS Inc., Chicago, IL, USA). The markers of bone turnover followed a logarithmically normal distribution and were expressed as the geometrical mean and SD. The mean values of different parameters from different groups were compared with each other for significant differences and were assessed using one-way analysis of variance (ANOVA) whenever significant differences were found. Correlations between BMD at different skeletal sites and bone turnover biomarker levels were made using Pearson’s correlation coefficient and a partial correlation analysis after adjustments for age and body mass index. Multivariate linear stepwise analysis was performed to determine how much of the variance in BMD T-scores at different skeletal sites was attributable to variations in bone biomarker levels. According to the World Health Organization (WHO) definitions [29] and the BMD reference databases established by our group [30], subjects with BMD of 2.5 SD lower (T-scores ≤ −2.5) than the peak BMD of the same gender were determined to be osteoporosis. The *χ*^2^ test was used to compare the prevalence of osteoporosis in different quartile groups.

## Results

### Age-related characteristics and parameters

Table 1 shows the age-related basic characteristics, bone turnover markers and BMD T-scores of the study subjects. The height and urinary creatinine of the subjects were markedly greater and the body mass index was notably lower in subjects aged 20–29 years and 30–39 years than in the other subjects. The levels of bone turnover markers also changed with age. All bone turnover marker levels were lowest in women aged 30–39 years, the levels of BAP, OC, uNTX, uCTX and uDPD increased significantly in women aged 40–49 years, and all levels increased further still in women aged over 50 years. The T-scores of the BMDs at various skeletal sites reduced significantly after the age of 50–59 years. The T-scores in women aged 60–69 years and 70 or more years were remarkably lower than the scores in all other age groups.

**Table 1:** Age-related characteristics, bone turnover markers and T-scores at various skeletal sites

**Marker**	**Age (years)**					
	**20–29 (*n*** **= 124)**	**30–39 (*n*** **= 141)**	**40–49 (*n*** **= 238)**	**50–59 (*n*** **= 229)**	**60–69 (*n*** **= 133)**	**≥70 (*n*** **= 26)**
**Height (cm)**	158 ± 5.6^b^	157 ± 5.3^b^	155 ± 5.1	154 ± 4.6	151 ± 5.0	152 ± 4.6
**Weight (kg)**	50.1 ± 6.5^b^	54.3 ± 7.4	57.5 ± 8.1	56.1 ± 8.5	54.5 ± 8.7	54.4 ± 9.3
**BMI (kg/m**^**2**^**)**	20.2 ± 2.3^b^	22.0 ± 2.5^b^	23.9 ± 3.0	23.7 ± 3.3	23.7 ± 3.2	23.7 ± 3.7
**MSU-Cr (mmol/L)**	11.4 ± 5.1^b^	11.0 ± 5.0^b^	8.8 ± 4.7	7.7 ± 4.3	7.8 ± 4.4	8.9 ± 5.2
**BAP (U/L)**^**a**^	16.0 ± 1.3	15.5 ± 1.4	22.0 ± 1.5^c^	32.1 ± 1.4^d^	29.1 ± 1.4	25.5 ± 1.4
**OC (μg/L)**^**a**^	7.3 ± 1.5	4.7 ± 1.6	5.7 ± 1.8^c^	10.6 ± 1.6^d^	10.4 ± 1.6	10.2 ± 1.4
**sNTX (nmol/L)**^**a**^	13.9 ± 1.3	13.0 ± 1.3	13.2 ± 1.5	16.2 ± 1.5^d^	18.0 ± 1.5	19.0 ± 1.3
**sCTX (mg/L)**^**a**^	3.7 ± 1.4	2.4 ± 1.6	2.6 ± 1.9	4.1 ± 1.6^d^	4.1 ± 1.6	4.9 ± 1.6
**uNTX (nM BCE/mM Cr)**^**a**^	33.3 ± 1.6	24.6 ± 1.7	33.3 ± 2.2^c^	60.3 ± 2.2^d^	49.8 ± 2.2	36.4 ± 1.9
**uCTX (μg/mM Cr)**^**a**^	167 ± 1.7	85.1 ± 3.0	117 ± 2.6^c^	244 ± 2.2^d^	235 ± 2.1	193 ± 2.1
**uDPD (nM/mM Cr)**^**a**^	6.1 ± 1.4	4.2 ± 1.8	5.0 ± 1.8^c^	6.3 ± 1.6^d^	6.1 ± 1.7	5.9 ± 1.6
**PA T-score**	0.0 ± 0.8	0.1 ± 0.9	−0.2 ± 1.1	−1.2 ± 1.1^d^	−2.0 ± 1.0^e^	−2.1 ± 1.0^e^
**FN T-score**	−0.1 ± 1.1	−0.1 ± 1.2	−0.2 ± 1.2	−1.1 ± 1.2^d^	−2.0 ± 1.0^e^	−2.4 ± 1.0^e^
**Hip T-score**	0.1 ± 0.9	0.1 ± 0.9	0.0 ± 1.0	−0.7 ± 0.9^d^	−1.5 ± 0.8^e^	−1.8 ± 1.0^e^

*Abbreviations*: *BMI* body mass index, *MSU* Cr-morning second urinary creatinine, *BAP* bone-specific alkaline phosphatase, *OC* osteocalcin, *sNTX* serum cross-linked N-terminal telopeptides of type I collagen (NTX), *sCTX* serum cross-linked C-terminal telopeptides of type I collagen (CTX), *uNTX* urinary NTX, *uCTX* urinary CTX, *uDPD* urinary deoxypyridinoline, *Cr* reatinine, *BCE* bone collagen equivalents, *PA* posteroanterior spine, *FN* femoral neck, *Hip* total hip. ^a^Values are expressed as geometric mean ± SD. ^b^*P* = 0.005–0.000 compared with various age groups of ≥40 years. ^c^*P* = 0.032–0.000 compared with the age groups of 30–39 years. ^d^*P* = 0.025–0.000 compared with the age groups of 30–39 and 40–49 years. ^e^*P* = 0.000 compared with various age groups of <60 years.

### Correlation between markers and BMD

Table 2 shows in premenopausal and postmenopausal women after adjusting the height and weight, the correlation coefficients between BMD and various bone turnover markers. Whether in premenopausal or postmenopausal women, PA lumbar spine BMD and bone turnover markers related degree is always better than the FN-BMD and Hip-BMD. In postmenopausal women, FN-BMD and Hip-BMD were negatively related to the level of OC, and showed no significant correlation with other bone turnover markers. In all of the subjects, there is a negative relationship between various bone turnover markers and the BMD T-score (all *P* = 0.038–0.000) of PA lumbar spine (*r* = −0.17 to −0.52), femoral neck (*r* = −0.08 to −0.44) and Hip (*r* = −0.11 to −0.46). Of these, the correlation between serum OC levels and T-scores at various skeletal sites was consistently the strongest. After adjustments for age and BMI, the partial correlation coefficients were markedly lower than the Pearson’s correlation coefficients. Nonetheless, the partial correlation coefficients of all bone turnover markers, except for uNTX and uDPD, with the T-scores at various skeletal sites were significant.

**Table 2:** Coefficients of correlation between BMD at various skeletal sites and bone turnover markers corrected for height and weight in the pre- and postmenopausal women

**Marker**	**Premenopausal**			**Postmenopausal**		
	**PA-BMD**	**FN-BMD**	**Hip-BMD**	**PA-BMD**	**FN-BMD**	**Hip-BMD**
**BAP**	−0.35*	−0.16^▲^	−0.21*	−0.30*	−0.01	−0.05
**OC**	−0.27*	−0.11^▲^	−0.12^▲^	−0.30*	−0.19^▲^	−0.22*
**sNTX**	−0.15^▲^	−0.11^▲^	−0.08	−0.02	−0.06	−0.08
**sCTX**	−0.20*	−0.14^▲^	−0.07	−0.23*	−0.10	−0.10
**uNTX**	−0.05	−0.06	−0.10	−0.09	0.02	−0.01
**uCTX**	−0.16^▲^	−0.08	−0.07	−0.14^▲^	−0.01	−0.06
**uDPD**	−0.10	−0.05	−0.03	−0.05	0.10	0.04

*Abbreviations*: as in Table 1. *P = 0.000. ^▲^*P* = 0.040–0.003.

### Association between markers and T-scores

**Figure 1:**
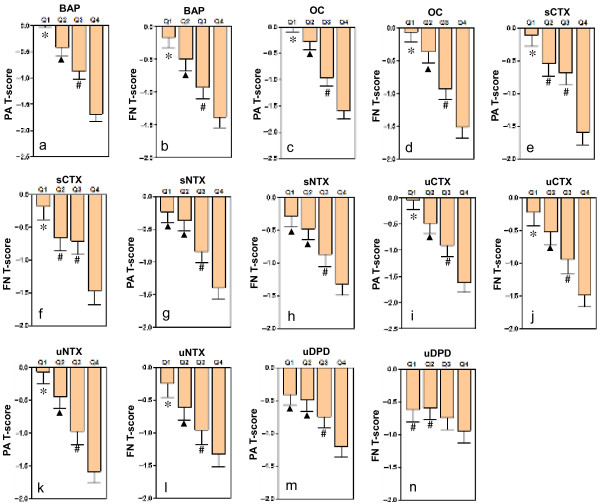
**BMD T-scores (mean and SE) at different skeletal sites for native Chinese women, by quartiles of bone turnover markers.** Abbreviations: BAP-bone-specific alkaline phosphatase, OC-osteocalcin, sCTX-serum cross-linked C-terminal telopeptides of type I collagen (CTX), sNTX-serum cross-linked N-terminal telopeptides of type I collagen, uCTX-urinary CTX, uNTX-urinary NTX, uDPD-urinary deoxypyridinoline, PA-posteroanterior spine, FN-femoral neck, Q1-first quartile, Q2-second quartile, Q3-third quartile, Q4-fourth quartile. *P = 0.045–0.000 compared with Q2, Q3 and Q4. ^▲^*P* = 0.029–0.000 compared with Q3 and Q4. ^#^*P* = 0.013–0.000 compared with Q4.

Figure 1 shows the grouping of the levels of bone turnover markers according to quartile intervals, and a comparison of the BMD T-scores in the different study groups. A marked, progressive decrease of T-scores at various skeletal sites was observed with increase in bone turnover marker levels. Apart from sNTX, sCTX and uDPD, the remaining bone turnover markers were associated with an decreasing tendency of T-scores at various skeletal sites in the order of Q1 > Q2 > Q3 > Q4. By using bone turnover markers as independent variables and T-scores as dependent variables, we performed a multiple linear regression stepwise analysis (Table 3). The results showed that serum BAP and OC were significant negative determinants of the T-score of the PA spine, and accounted for approximately 33.1% and 7.8% of the changes in T-scores of the PA spine. Serum OC, BAP and sNTX were significant negative determinants of T-scores at femoral neck and total hip.

**Table 3:** Multiple linear regression analysis of markers of bone turnover and BMD T-scores at various skeletal sites

**Independent**	**PA T-score**		**FN T-score**		**Hip T-score**	
	***β***	**Adjusted *R***^**2 **^**C (%)**	***β***	**Adjusted *R***^**2 **^**C (%)**	***β***	**Adjusted *R***^**2 **^**C (%)**
**BAP**	−0.39*	33.1	−0.20*	2.8	−0.22*	3.3
**OC**	−0.34*	7.8	−0.35*	20.4	−0.35*	21.9
**sNTX**	―^▲^	―^▲^	−0.10*	0.7	−0.08*	0.4
**sCTX**	―^▲^	―^▲^	―^▲^	―^▲^	―^▲^	―^▲^
**uNTX**	―^▲^	―^▲^	―^▲^	―^▲^	―^▲^	―^▲^
**uCTX**	―^▲^	―^▲^	―^▲^	―^▲^	―^▲^	―^▲^
**uDPD**	―^▲^	―^▲^	0.11*	0.8	0.09*	0.5

Bone turnover markers are identified as independent variables, while BMD T-scores at different skeletal sites are identified as dependent variables. *Abbreviations*: as in Table 1, R^2^ C(%)-R square change. *P = 0.040–0.000. ^▲^Marker was excluded from this analysis.

### Relationship between markers and the risk of osteoporosis

**Figure 2:**
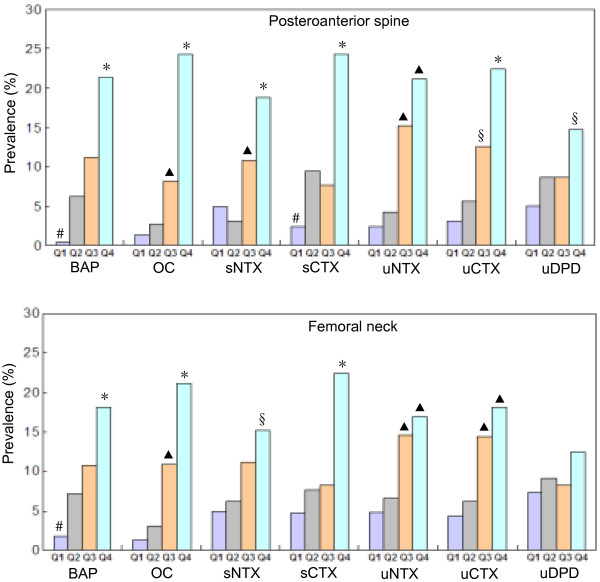
**Comparison of prevalence of osteoporosis diagnosed using BMD T-scores at different skeletal sites in native Chinese women, by quartiles of bone turnover markers.** Abbreviations: as in Figure 1. *P = 0.037–0.000 compared with Q1, Q2 and Q3. ^▲^*P* = 0.035–0.000 compared with Q1 and Q2. ^#^*P* = 0.048–0.000 compared with Q2 and Q3. ^§^*P* = 0.024–0.001 compared with Q1.

Figure 2 shows the grouping of bone turnover markers according to quartile intervals and a comparison of the prevalence of osteoporosis among different groups. At the PA lumbar spine, when BAP, OC, sNTX, sCTX and uCTX were grouped, the prevalence of osteoporosis in Q4 group was significantly higher than those in Q1, Q2 and Q3 groups; while, the prevalences of osteoporosis in Q1 and Q2 groups were significantly lower than Q3 group when OC, sNTX and uCTX were grouped. At the femoral neck, when BAP, OC and sCTX were grouped by quartile, the prevalence of osteoporosis in Q4 group was significantly higher than those in Q1, Q2 and Q3 groups; when OC, uNTX and uCTX were grouped, the prevalences of osteoporosis in Q1 and Q2 groups were significantly lower than Q3 group. But there were no significant differences between each group when uDPD was grouped according to quartile.

Table 4 shows the effects of the changes in bone turnover marker levels on the risks of osteoporosis. The risks for osteoporosis at various skeletal sites increased with the levels of bone turnover markers. We calculated odds ratios (ORs) by using the highest bone turnover marker level in the Q4 groups (case groups) and the lowest level in the Q1 groups (control groups). In the case of the BAP level, the risk of osteoporosis of the PA spine and femoral neck in the Q4 group was 13 times and 4.8 times the risk in the Q1 group, respectively. The corresponding osteoporotic risks for the OC levels were 11 times and 7.8 times, whereas those for the sNTX levels were 4.5 times and 3.5 times.

**Table 4:** Odds ratio (OR) and 95% confidence intervals (CI) of osteoporosis diagnosed using BMD T-scores at different skeletal sites in various quartiles (Q) of bone turnover markers

	*Q4/Q1^▲^	*Q3/Q1^▲^	*Q4/Q2^▲^	*Q3/Q2^▲^
	**OR (95% CI)**	**OR (95% CI)**	**OR (95% CI)**	**OR (95% CI)**
**BAP categories**				
**PA**	13 (6.4–19)	7.8 (3.5–16)	4.1 (2.2–7.6)	1.9 (1.0–3.7)
**FN**	4.8 (3.3–14)	5.9 (2.2–15)	2.9 (1.6–5.3)	1.6 (0.8–3.0)
**OC categories**				
**PA**	11 (6.1–18)	5.8 (1.9–13)	6.2 (4.1–17)	3.2 (1.2–8.2)
**FN**	7.8 (4.9–15)	3.9 (2.6–10)	5.3 (3.2–16)	3.7 (1.6–8.9)
**sNTX categories**				
**PA**	4.5 (2.2–8.9)	2.3 (1.1–4.9)	7.2 (3.2–16)	3.7 (1.6–8.9)
**FN**	3.5 (1.7–7.0)	2.4 (1.2–5.2)	2.7 (1.4–5.2)	1.9 (1.0–3.7)
**sCTX categories**				
**PA**	6.7 (4.6–18)	3.4 (1.1–11)	3.1 (1.7–5.8)	0.8 (0.4–1.7)
**FN**	5.8 (2.6–13)	1.8 (0.7–4.4)	3.5 (1.8–6.9)	1.1 (0.5–2.4)
**uNTX categories**				
**PA**	8.9 (3.8–16)	4.9 (2.4–14)	5.1 (2.6–12)	4.0 (1.7–9.6)
**FN**	4.0 (1.8–9.1)	3.3 (1.5–7.7)	2.9 (1.4–5.9)	2.4 (1.1–5.0)
**uCTX categories**				
**PA**	8.4 (3.4–15)	4.4 (1.6–12)	4.9 (2.3–10)	2.4 (1.1–5.4)
**FN**	4.8 (2.1–11)	3.7 (1.5–8.8)	3.3 (1.6–7.1)	2.5 (1.2–5.5)
**uDPD categories**				
**PA**	3.2 (1.6–6.6)	1.8 (0.8–3.9)	1.8 (1.0–3.3)	1.0 (0.5–1.9)
**FN**	1.8 (0.9–3.4)	1.1 (0.6–2.3)	1.4 (0.8–2.6)	0.9 (0.5–1.7)

*As the case group, ^▲^as the control group to calculate ORs. *Abbreviations*: as in Table 1 and Figure 1.

## Discussion

Our research confirmed the significant negative correlation between the levels of bone turnover markers with BMD and T-scores at various skeletal sites in native Chinese women; in other words, the BMD and T-scores decreased as the levels of various bone turnover markers increased. The best correlation (consistent maximum *r* values) of bone turnover markers was seen with the BMD and T-score of the PA spine. After adjustments for age and BMI, the partial correlation coefficients between bone turnover markers and T-scores were lower than the Pearson’s correlation coefficients. With the exception of uNTX and uDPD, all other bone turnover markers showed statistically significant partial correlation coefficients with T-scores. These results indicate that a varying degree of correlation is present between individual bone turnover markers and the T-scores at different skeletal sites. After excluding the influence of age and BMI, this correlation might reduce. After adjustment of height and weight, BMD in premenopausal and postmenopausal women showed significant negative correlation with bone turnover markers, which was similar to the results of previous studies [31]. Other studies have verified that changes in the levels of bone turnover markers are relevant to bone loss rates in the lumbar vertebrae and distal end of the forearm [6,13,17,32-38], but not relevant to bone loss rates in the hip [9,39]. Keen et al. [14] showed that bone turnover markers are not related to the bone loss rates in the lumbar spine and the femoral neck. Moreover, after adjustments for BMI, bone turnover markers, including serum OC and uNTX, were not related to bone loss rates in both the lumbar spine and femoral neck in aged women [39]. In addition, studies have shown that bone turnover markers can predict the bone loss rate during the period of transition to menopause, but not after menopause [15].

We grouped the levels of bone turnover markers using quartile intervals and confirmed that the average T-scores significantly differ among different groups. In the case of BAP, OC, uNTX and uCTX, progressive increase in marker levels was associated with a marked and progressive decrease in the average T-scores of the lumbar spine and femoral neck (Q1 > Q2 > Q3 > Q4). In the case of the bone resorption markers, the average T-scores did not significantly differ between the Q1 and Q2 groups of sNTX and uDPD or between the Q2 and Q3 groups of sCTX. These results verified that the levels of bone turnover markers, particularly BAP, OC, uNTX and uCTX, and the T-scores at various skeletal sites were closely correlated in native Chinese women. Studies in other populations have shown that the rate of bone loss is related to an increase in bone turnover markers in women [40]. When the levels of bone turnover markers increased by 1 SD, the risk of rapid bone loss (2.2% per year) increased by 1.8–2.0 times. In subjects in whom the serum BAP level was 2 SD more than the average level, the probability of rapid bone loss was 80% [40]. However, when the serum BAP level was 2 SD less than the average level, this probability was only 20%.

Multiple linear regression stepwise analysis was used to compare the effects of the changes in the levels of various bone turnover markers on the BMD T-scores at various skeletal sites. Using the T-scores as dependent variables and bone turnover markers as independent variables, we found that changes in levels of BAP and OC significantly influenced the T-scores of the PA spine; these levels jointly accounted for 40.9% of the variations in the T-scores of the PA spine. Of these markers, BAP had the largest influence on the T-scores of the PA spine (33.1%). None of the bone resorption markers had any effect on the T-scores of the PA spine. Serum OC, BAP and sNTX jointly accounted for 23.9% and 25.6% of the variations in the femoral neck and total hip T-scores, respectively. Of these markers, serum OC had the largest influence on the femoral neck (20.4%) and total hip T-scores (21.9%). sCTX, uNTX and uCTX had no effect on the femoral neck and total hip T-scores. Serum BAP was the greatest determinant of the T-scores of the PA spine, but serum OC was an important predictive factor of the femoral neck and total hip T-scores. The reasons underlying these findings might be related to differences in the compositions of cortical and cancellous bones. The turnover of cancellous bones is eight times faster than that of cortical bones [41]. Other studies have reported that serum OC, uNTX and serum parathyroid hormone (PTH) jointly account for 43% of the decrease in total hip BMD in aged women [11]. The combination of bone resorption markers and bone formation markers or the combination of pyridinoline, estrogen and BMI could predict 59% of changes in the bone loss rate of the forearm [37,42].

Our study confirmed that increase in bone turnover marker levels was associated with an increase in the prevalence and risk of osteoporosis in native Chinese women. These results show that individuals with increased bone turnover marker levels, especially BAP, OC, uNTX and uCTX, have a higher prevalence of osteoporosis. We considered subjects with high bone turnover marker levels as the case group and those with low levels as the control group, and calculated that the risk of osteoporosis increased with higher levels of bone turnover markers. This risk was different for different bone turnover markers and skeletal sites. For all bone turnover markers other than uDPD, the risk of osteoporosis of the PA lumbar spine was 4.5–13 times greater in the Q4 group than in the Q1 group. In the case of the femoral neck, this difference was 3.5–7.8 times. These results also suggest that the risk of osteoporosis greatly increases with increase in the levels of bone turnover markers in native Chinese women. In addition, it is worth pointing out the limitations of this study, even though the second morning urine creatinine levels of these subjects were in the reference value range, but we did not have the simultaneous determination of their serum creatinine levels and glomerular filtration rate (eGFR), to investigate the relationship between bone turnover markers and eGFR in this study population. Because the renal function is closely associated with bone metabolism and osteoporosis.

## Conclusions

This study revealed a close correlation between bone turnover markers and BMD at various skeletal sites in native Chinese women. The levels of bone turnover markers determine the T-scores at various skeletal sites, and increase in the levels of these markers was associated with a significantly increased risk of osteoporosis. Markers of bone formation, including serum BAP and OC, were the main determinants of T-scores.

## Abbreviations

BMD: Bone mineral density; OC: Osteocalcin; BAP: Bone-specific alkaline phosphatase; sNTX: Serum cross-linked N-terminal telopeptides of type I collagen; sCTX: Serum cross-linked C-terminal telopeptides of type I collagen; uNTX: Urinary cross-linked N-terminal telopeptides of type I collagen; uCTX: Urinary cross-linked C-terminal telopeptides of type I collagen; uDPD: Urinary deoxypyridinoline; Cr: Reatinine; ELISA: Enzyme-linked immunosorbent assay; BCE: Bone collagen equivalents; PA: Posteroanterior spine; FN: Femoral neck; BMI: Body mass index; DXA: Dual energy x-ray absorptiometry; WHO: World Health Organization; R^2^C: R square change; Q1: First quartile; Q2: Second quartile; Q3: Third quartile; Q4: Fourth quartile; OR: Odds ratio.

## Competing interests

The authors declare that they have no conflict of interests.

## Authors’ contributions

Study design, data analysis, and data interpretation: XYW, HLL, HX, XHL, YQP, LQY, ZFS, RCD, XPW and EYL. Drafting manuscript and revising manuscript content: XYW, XPW, and EYL. Approving final version of manuscript: XYW, HLL, XPW and EYL. All authors read and approved the final manuscript.

## Pre-publication history

The pre-publication history for this paper can be accessed here:

http://www.biomedcentral.com/1472-6823/14/8/prepub
